# Carbonyl Compounds Generated from Electronic Cigarettes

**DOI:** 10.3390/ijerph111111192

**Published:** 2014-10-28

**Authors:** Kanae Bekki, Shigehisa Uchiyama, Kazushi Ohta, Yohei Inaba, Hideki Nakagome, Naoki Kunugita

**Affiliations:** 1 Department of Environmental Health, National Institute of Public Health, 2-3-6 Minami, Wako-shi, Saitama 351-0197, Japan; E-Mails: bekki.kanae@niph.go.jp (K.B.); yohei_inaba@niph.go.jp (Y.I.); kunugita@niph.go.jp (N.K.); 2 Graduated School of Engineering, Chiba University, 1-33 Yayoi-cho, Inage-ku, Chiba-shi, Chiba 263-8522, Japan; E-Mails: uchiyama.s@chiba-u.jp (K.O.); nakagome@tu.chiba-u.ac.jp (H.N.)

**Keywords:** electronic cigarette, e-cigarette aerosol, carbonyl compounds

## Abstract

Electronic cigarettes (e-cigarettes) are advertised as being safer than tobacco cigarettes products as the chemical compounds inhaled from e-cigarettes are believed to be fewer and less toxic than those from tobacco cigarettes. Therefore, continuous careful monitoring and risk management of e-cigarettes should be implemented, with the aim of protecting and promoting public health worldwide. Moreover, basic scientific data are required for the regulation of e-cigarette. To date, there have been reports of many hazardous chemical compounds generated from e-cigarettes, particularly carbonyl compounds such as formaldehyde, acetaldehyde, acrolein, and glyoxal, which are often found in e-cigarette aerosols. These carbonyl compounds are incidentally generated by the oxidation of e-liquid (liquid in e-cigarette; glycerol and glycols) when the liquid comes in contact with the heated nichrome wire. The compositions and concentrations of these compounds vary depending on the type of e-liquid and the battery voltage. In some cases, extremely high concentrations of these carbonyl compounds are generated, and may contribute to various health effects. Suppliers, risk management organizations, and users of e-cigarettes should be aware of this phenomenon.

## 1. Introduction

An electronic cigarette (e-cigarette) is a battery-powered device designed to deliver nicotine to a smoker. It was first developed by Herbert A. Gilbert, who patented a device described as “a smokeless non-tobacco cigarette” that involved “replacing burning tobacco and paper with heated, moist, flavored air” in 1963 [1]. However, the invention of the e-cigarette in 2003 is attributed to Hon Lik, a Chinese pharmacist, and e-cigarettes were introduced to the Chinese market as a smoking cessation device in 2004 [2]. There are several types of e-cigarettes, which include nicotine or are nicotine-free liquid-holding cartridges. E-cigarettes are presented as low-risk products, with a realistic look, feel, and taste when compared with traditional cigarettes [3]. Among major carcinogens and toxic compounds such as nitrosamines and polycyclic aromatic hydrocarbons (PAHs) in traditional cigarette smoke, several combustion products are included in the e-cigarette aerosol, too. Nitrosamines are present at levels almost similar to nicotine replacement therapies (NRTs) [4], and PAHs are completely absent from e-cigarettes. E-cigarette vendors have marketed their products as a cheaper and safer smokeless alternative to traditional cigarettes and a possible smoking cessation tool. Consequently, many cigarette smokers have turned to e-cigarettes, and the number of e-cigarette smokers is increasing [5,6,7]. According to a report by UBS Securities LLC (Union Bank of Switzerland, Zurich, Switzerland), e-cigarette market sales doubled from $250–$500 million between 2011 and 2012 and are expected to quadruple by 2014 [8]. In recent years, on the international market, e-cigarettes have been widely advertised via television, radio, magazines, newspapers, and the Internet. This mass marketing and commercialization of e-cigarettes is estimated to increase consumer awareness and the future use of e-cigarettes [9]. Additionally, the legal situation may be contributing to the widespread use of e-cigarettes. The World Health Organization (WHO) raised the alarm with regard to e-cigarettes that include nicotine and issued a Technical Report Series 955 in 2009 which states the following: the safety of e-cigarettes is not confirmed, and e-cigarettes are not an appropriate tool for smoking cessation therapy [10]. The Food and Drug Administration (FDA) reported that e-cigarettes contain carcinogens and toxic chemicals, such as nitrosamines and diethylene glycol, which have potentially harmful effects on humans [11]. Furthermore, the FDA found that nicotine was detected in the e-cigarette cartridges labeled as nicotine-free [10,12], and carcinogens and toxic chemicals, such as carbonyl compounds, were detected in the aerosols from e-cigarettes [7,13,14]. Evaluating the source and amount of carbonyl compounds released is crucial for regulators as well as consumers and manufacturers, and ways to reduce such emissions need to be investigated. This paper presents an overview of our research in this field, as well as a comparison with other relevant studies. 

In this article, we review the results of our research over the past four years, and incorporate the current literature found in Science Direct, PubMed, and Google Scholar databases from journal articles published between 2010 and 2014. Various combinations of keywords, such as “e-cigarette”, “electronic cigarette”, “chemical components” and “carbonyl compounds” were used to find the relevant literature.

## 2. Carbonyl Compounds Emitted from Japanese E-Cigarettes

Uchiyama *et al.* measured carbonyl compounds in e-cigarette aerosols using cartridges impregnated with hydroquinone (HQ) and 2,4-dinitrophenylhydrazine (DNPH), followed by high-performance liquid chromatography (HPLC) [13,14,15]. Before collecting the aerosol from the e-cigarettes, an HQ-cartridge and a DNPH-cartridge were connected. The coupled cartridges were then connected between the mouthpiece of the e-cigarette and the smoking machine, and the aerosol from the e-cigarette was drawn into the coupled cartridges (from the HQ-cartridge to the DNPH-cartridge) according to the Canadian intense regimen (55 mL puff volume, 2-s puff duration, 30-s puff interval, and 10 puffs) [16]. After collection, the coupled cartridges were extracted using acetonitrile containing 1% phosphoric acid in the direction opposite to that of the air sampling until a 4.5 mL total volume was attained. After 10 min, ethanol (0.5 mL) was added to the eluate, and the solution was analyzed by HPLC.

Thirteen brands of Japanese e-cigarettes were measured, and several derivative peaks of carbonyl compounds, such as formaldehyde, acetaldehyde, acetone, acrolein, propanal, crotonaldehyde, butanal, glyoxal, and methylglyoxal, were detected [13,14]. Table 1 shows the mean amounts of the major carbonyl compounds generated from the Japanese e-cigarettes. For a typical cigarette smoking experience of 10 puffs, these values were translated into μg/10 puffs. The top entry in each cell indicates the mean value for the high-amount group, and the bottom entry indicates the mean value for the low-amount group. The indices *N*_high_ and *N*_low_ indicate the number of e-cigarettes that generated high and low concentrations of carbonyl compounds, respectively. As described below, carbonyl compounds were incidentally generated by touching the nichrome wire with e-liquid and increasing the battery output voltage. Therefore, concentrations showed bimodal distributions and were divided in extremely high and low groups. For clarity, the failure rate (FR) is indicated in Table 1. FR was calculated by the following equation: FR = *N*_high_/(*N*_high_ + *N*_low_) × 100. 

**Table 1:** Amounts (μg/10 puff) of major carbonyl compounds generated from 13 brands of Japanese e-cigarettes. Smoking machine was performed at 10 puffs (reproduced from [13] with permission from The Japan Society for Analytical Chemistry).

Product	N_high_ N_low_	FR	Formaldehyde	Acetaldehyde	Acrolein	Propanal	Glyoxal	Methylglyoxal
A	16 35	31	34 ± 35 n.d.	26 ± 28 n.d.	4.1 ± 3.8 n.d.	8.8 ± 11 n.d.	2.5 ± 3.6 n.d.	2.9 ± 3.1 n.d.
B	06 24	20	13 ± 5.8 1.4 ± 0.9	0.2 ± 0.1 n.d.	6.6 ± 2.4 1.2 ± 0.9	1.1 ± 0.7 n.d.	16± 6.6 n.d.	11 ± 4.3 2.0 ± 1.2
C	08 22	27	22 ± 15.4 1.7 ± 1.4	0.9 ± 1.4 n.d.	5.3 ± 5.5 0.6 ± 0.6	3.4 ± 3.5 n.d.	9.9 ± 5.2 0.7 ± 0.7	12.1 ± 5.5 1.2 ± 1.0
D	12 37	24	15 ± 6.6 0.8 ± 1.0	13.8 ± 6.6 n.d.	20 ± 9.9 n.d.	13.2 ± 10.5 n.d.	4.2 ± 2.3 n.d.	6.1 ± 4.1 n.d.
E	14 21	40	17 ± 7.7 0.7 ± 0.8	15 ± 6.1 n.d.	18 ± 6.6 0.7 ± 0.9	15 ± 8.3 n.d.	4.5 ± 2.4 n.d.	4.7 ± 4.3 n.d.
F	02 03	40	6.6 ± 0.9 2.0 ± 1.7	1.5 ± 0.1 0.9 ± 0.2	1.1 ± 0.1 0.7 ± 0.3	0.4 ± 0.1 n.d.	1.5 ± 0.4 n.d.	3.2 ± 0.5 0.9 ± 0.8
G	01 25	4	29 n.d.	10 n.d.	10 n.d.	3.5 n.d.	9.4 n.d.	20 n.d.
H	05 25	17	10 ± 4.9 0.9 ± 1.4	4.6 ± 2.4 n.d.	4.5 ± 2.2 n.d.	n.d. n.d.	2.5 ± 0.5 n.d.	4.6 ± 3.1 n.d.
I	06 24	20	3.2 ± 1.0 1.5 ± 1.4	6.1 ± 3.2 2.6 ± 2.9	6.1 ± 2.3 2.8 ± 2.7	7.7 ± 2.3 3.3 ± 3.4	n.d. n.d.	n.d. n.d.
J	00 04	0	n.a. n.d.	n.a. n.d.	n.a. n.d.	n.a. n.d.	n.a. n.d.	n.a. n.d.
K	00 30	0	n.a. n.d.	n.a. n.d.	n.a. n.d.	n.a. n.d.	n.a. n.d.	n.a. n.d.
L	00 30	0	n.a. n.d.	n.a. n.d.	n.a. n.d.	n.a. n.d.	n.a. n.d.	n.a. n.d.
M	00 13	0	n.a. n.d.	n.a. n.d.	n.a. n.d.	n.a. n.d.	n.a. n.d.	n.a. n.d.

Notes: Resulting data were divided into two groups based on the formaldehyde concentration (10 mg/m^3^). The upper line indicates the mean value for the high-concentration group, and the lower line indicates the mean value for the low-amount group. Indices *N*_high_ and *N*_low_ indicate the number of e-cigarettes that generated high and low amounts of carbonyl compounds, respectively. FR indicates the failure rate, which was calculated using the equation as follows: FR = *N*_high_/(*N*_high_ + *N*_low_) × 100. Values are mean ± SD; n.a., not available; n.d., not detected.

Four (J, K, L, and M) out of the 13 e-cigarette brands did not generate any carbonyl compounds. The other nine e-cigarette brands (A, B, C, D, E, F, G, H, and I) generated various carbonyl compounds. The amount of carbonyl compounds obtained for the high-amount group was significantly higher than that obtained for the low-amount group. The maximum concentrations of formaldehyde, acetaldehyde, acrolein, propanal, glyoxal, and methylglyoxal were 140, 120, 40, 46, 23, and 21 μg/10 puffs, respectively. Most notably, very high amounts of formaldehyde were measured in e-cigarette aerosols. Glyoxal and methylglyoxal, which show mutagenicity, are specific to e-cigarette aerosols and have been minimally detected in the mainstream smoke from traditional cigarettes. The amount of carbonyl compounds in these brands of e-cigarettes varied significantly not only among different brands but also among different samples of the same products. 

## 3. Mechanism for Generation of Carbonyl Compounds from E-Cigarettes 

**Figure 1:**
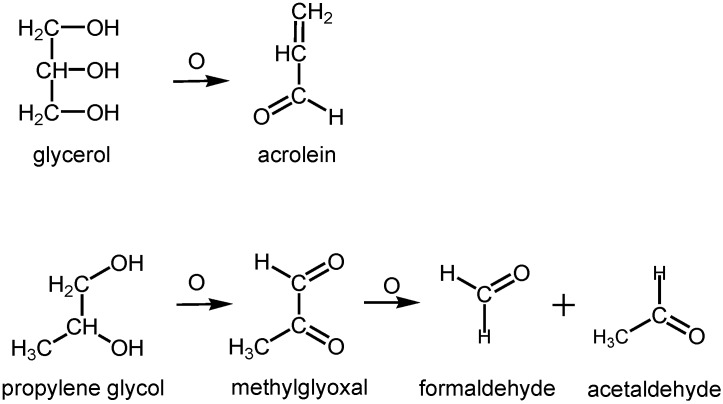
Oxidation of e-liquid (glycerol and propylene glycol) with nichrome wire (reproduced from [14] with permission from The Japan Society for Analytical Chemistry).

The design of most e-cigarettes includes a plastic tube holding a battery, an air flow sensor, a vaporizer, and a nicotine/flavor cartridge with a chemical component, such as glycerols or glycols, which turn the liquid to aerosol [17]. The function of e-cigarettes has changed from disposable and rechargeable to “tank systems” that can hold a large volume of e-liquid. This e-liquid incidentally touches the heated nichrome wire and is oxidized to formaldehyde, acetaldehyde, acrolein, glyoxal, and methylglyoxal in the presence of oxygen in the surrounding air [13,14]. There is a great variety of commercial e-liquids manufactured and distributed by various companies. Figure 1 shows the reaction of e-liquid with heated nichrome wire in the study of Uchiyama *et al.* [13] and Ohta *et al.* [14]. Glycerol in e-liquid is oxidized with heated nichrome wire to form acrolein, while propylene glycol in e-liquid is oxidized to form methyl glyoxal, formaldehyde, and acetaldehyde [13,14]. This e-liquid inadvertently touches the heated nichrome wire to form these oxidation products.

Furthermore, battery output voltage affects the concentration of the carbonyl compounds in the emission [7]. Some new e-cigarettes allow users to increase vapor production and nicotine delivery by changing the battery output voltage. Kosmider *et al.* showed that increasing the voltage from 3.2–4.8 V resulted in a 4 to >200 times increase in the formaldehyde, acetaldehyde, and acetone levels [7]. In fact, the levels of formaldehyde in vapors from high-voltage devices were almost identical to those in traditional cigarette smoke (1.6–52 μg per cigarette) [18]. Ohta *et al.* further reported that increasing levels of carbonyl compounds, such as formaldehyde and acetaldehyde, were observed for a voltage over 3 V [13,14]. Consequently, commercial e-cigarettes with 4–5 V batteries are sufficient to generate carbonyl compounds, with the battery output voltage significantly affecting the concentration of carbonyl compounds in the e-cigarette aerosol. As such, high-voltage e-cigarettes may expose users to high levels of carbonyl compounds.

## 4. Discussion 

E-cigarettes are advertised as less harmful products because they are believed to contain fewer and less toxic inhaled compounds than traditional cigarettes. Consequently, e-cigarettes are considered to be an appropriate tool for tobacco harm reduction, which describes actions taken to lower the health risks associated with using nicotine delivered through combustible tobacco [19]. However, e-cigarettes have not been theoretically or experimentally proven to be safer products. In fact, there are some case reports of health damage induced by e-cigarettes in many countries, including the USA and in Europe. The most common symptom is a dry mouth and throat [12,20] , which is considered to originate from the water-absorbing property of propylene glycol and glycerol, the main constituents of e-liquids. Furthermore, several health impacts such as hypertension, asthma, chronic obstructive pulmonary disease, lipoid pneumonia, cardiac arrhythmias, eosinophilic pneumonitis, congestive heart failure, disorientation, and hypotension are considered to be caused by e-cigarette use [21,22,23,24]. On the other hand, benefits of e-cigarette use such as smoking abstinence and a reduction in asthmatic smokers is reported in the survey of e-cigarette use [25]. 

However, regulation of the e-cigarette should be considered on the basis of reported adverse health effects. In some countries and regions, such as Europe and the USA, regulations regarding nicotine content, labeling, advertising, and sale of e-cigarettes are already in effect [26,27]. Some countries do not accept e-cigarettes as a cessation tool for smokers, yet regulate it as a medical product [28,29]. Recently, direction about manufacture, presentation, ingredients, sale, and certain aspects of labeling and packaging were adopted to regulate e-cigarette users and companies in Europe [30]. However, the chemical compounds generated from the e-cigarettes themselves are yet to be regulated. To promote e-cigarette regulation, we need to show more substantial scientific data about the impacts of e-cigarettes. In recent years, several studies, including those of our group, reported that carbonyl compounds such as formaldehyde, acetaldehyde, acrolein, and glyoxal are often found in e-cigarette aerosols, which are considered to have toxic effects on human health. Formaldehyde is classified as a human carcinogen (Group 1) by the International Agency for Research on Cancer (IARC), and acetaldehyde is classified as possible carcinogenic to humans (Group 2B) [31]. Acrolein causes irritation of the nasal cavity and damages the lining of the lungs [16]. These compounds in e-cigarettes are potentially hazardous and induce various health effects on its users. 

Some carbonyls, such as formaldehyde, acetaldehyde, and acrolein in e-cigarette emissions have also been reported in other countries [32,33,34]. Table 2 shows the amount of formaldehyde, acetaldehyde, and acrolein in the aerosols of Polish e-cigarettes [35]. According to these data, the emissions from e-cigarettes without propylene glycol were almost 100-fold lower than those from traditional cigarettes [36]. 

**Table 2:** Amounts (μg) of major carbonyl compounds generated from 12 brands of Polish e-cigarettes. Smoking machine was performed at 150 puffs (reproduced from [35] with permission from BMJ Publishing Group Ltd.).

Product	Formaldehyde	Acetaldehyde	Acrolein
EC01	44.2 ± 4.1	4.6 ± 0.2	41.9 ± 3.4
EC02	23.6 ± 8.7	6.8 ± 3.2	4.4 ± 2.5
EC03	30.2 ± 2.3	8.2 ± 2.5	16.6 ± 2.5
EC04	47.9 ± 0.2	11.5 ± 2.0	30.1 ± 6.4
EC05	56.1 ± 1.4	3.0 ± 0.2	22.0 ± 1.6
EC06	35.3 ± 2.7	13.6 ± 2.1	2.1 ± 0.4
EC07	19.0 ± 2.7	11.1 ± 3.3	8.5 ± 3.6
EC08	6.0 ± 2.0	8.8 ± 1.6	0.7 ± 0.4
EC09	3.2 ± 0.8	3.5 ± 0.3	ND
EC10	3.9 ± 1.5	2.0 ± 0.1	2.7 ± 1.6
EC11	23.9 ± 11.1	3.7 ± 1.5	1.1 ± 0.6
EC12	46.3 ± 2.1	12.0 ± 2.4	7.4 ± 3.2

Kosmider *et al.* reported that formaldehyde and acetaldehyde were detected in eight of 13 samples [7]. The amounts of formaldehyde and acetaldehyde in e-cigarette aerosols at a lower voltage were on average 13 and 807-fold lower than those in traditional cigarette smoke, respectively. The highest levels of carbonyls were observed in e-cigarette aerosols generated from propylene-glycol-based solutions. Furthermore, the data revealed large variations in carbonyl levels for different e-cigarettes. However, in general, there is an insufficient amount of data on the hazardous carbonyl compounds emitted from e-cigarettes, thus warranting continued broad monitoring of these compounds. 

## 5. Conclusions 

Studies have shown that e-cigarettes emit toxic carbonyl compounds, generated from thermal decomposition. These substances can have adverse health effects; however, in most cases, the levels are lower than those in tobacco cigarette smoke. It is important to expand the research in this field, to better understand the source of carbonyls emitted from e-cigarettes and find ways to reduce them. 
